# From fringe to centre-stage: experiences of mainstreaming health equity in a health research collaboration

**DOI:** 10.1186/s12961-020-00648-z

**Published:** 2021-03-03

**Authors:** Ana Porroche-Escudero, Jennie Popay, Fiona Ward, Saiqa Ahmed, Dorkas Akeju, Jane Cloke, Mark Gabbay, Shaima Hassan, Koser Khan, Esmaeil Khedmati-Morasae

**Affiliations:** 1 Lancaster Environment Centre, Lancaster University, Lancaster, UK; 2 Division of Health Research, Lancaster University, Lancaster, UK; 3 ARC NWC, Division of Health Research, Lancaster University, Lancaster, UK; 4 ARC NWC, Institute of Population Health, University of Liverpool, Liverpool, UK; 5 Business School, Exeter University, Exeter, UK

**Keywords:** Health inequalities, Mainstreaming, Research collaboration, Implementation, Social determinants of health, United Kingdom

## Abstract

**Background:**

Action to address the structural determinants of health inequalities is prioritized in high-level initiatives such as the United Nations Sustainable Development Goals and many national health strategies. Yet, the focus of much local policy and practice is on behaviour change. Research shows that whilst lifestyle approaches can improve population health, at best they fail to reduce health inequalities because they fail to address upstream structural determinants of behaviour and health outcomes. In health research, most efforts have been directed at three streams of work: understanding causal pathways; evaluating the equity impact of national policy; and developing and evaluating lifestyle/behavioural approaches to health improvement. As a result, there is a dearth of research on effective interventions to reduce health inequalities that can be developed and implemented at a local level.

**Objective:**

To describe an initiative that aimed to mainstream a focus on health equity in a large-scale research collaboration in the United Kingdom and to assess the impact on organizational culture, research processes and individual research practice.

**Methods:**

The study used multiple qualitative methods including semi-structured interviews, focus groups and workshops (*n* = 131 respondents including Public Advisers, university, National Health Service (NHS), and local and document review.

**Results:**

utilizing Extended Normalization Process Theory (ENPT) and gender mainstreaming theory, the evaluation illuminated (i) the processes developed by Collaboration for Leadership in Applied Health Research and Care North West Coast to integrate ways of thinking and acting to tackle the upstream social determinants of health inequities (i.e. to mainstream a health equity focus) and (ii) the factors that promoted or frustrated these efforts.

**Conclusions:**

Findings highlight the role of contextual factors and processes aimed at developing and implementing a robust strategy for mainstreaming health equity as building blocks for transformative change in applied health research.

## Introduction

Worldwide, health inequalities represent the main cause of lives lost prematurely as well as avoidable disability, suffering and distress [3, 9]. Efforts to understand and reduce these inequalities have a long history in the United Kingdom [11, 31], but the report of the World Health Organization (WHO)-sponsored Commission on the Social Determinants of Health in 2008 triggered a rapid expansion of both research and policy interest around the globe (European Portal for Action on Health Inequalities [14, 53]). Most notably, action to reduce health inequalities is prioritized in the United Nations Sustainable Development Goals endorsed by 193 nations in 2015 and in many national health strategies [7, 39]. Whilst these initiatives present promising opportunities to further integrate a focus on upstream social determinants of health inequalities in policy, practice, research and capacity-building activities, the primary focus for action continues to be on behaviour change [2, 36]. Research has shown that whilst lifestyle approaches may contribute to population health improvements overall, they are ineffective in reducing health inequalities, because the underlying structural causes are unchallenged [48]. Similarly, in health research, most efforts have been directed at three streams of work: understanding causal pathways; evaluating the equity impact of national policy on, for example, welfare benefits or housing; and developing and evaluating lifestyle/behavioural approaches to health improvement. As a result, there is a dearth of research-based evidence on effective interventions to reduce the upstream determinants of health inequalities that can be developed and implemented at a local level [2, 22], and published evidence about the processes and effectiveness of attempts is lacking (see [36]

The setting for this study is the National Institute for Health Research (NIHR) Collaboration for Leadership in Applied Health Research and Care North West Coast (CLAHRC NWC), a large English-based research and implementation partnership organization established in 2014. It aimed to contribute to reductions in health inequalities in North West England, which has some of the worse health in the United Kingdom. To do this it sought to embed a focus on reducing health inequalities into its organizational culture, research processes and activities, including evidence synthesis, applied research and implementation, capacity building and knowledge mobilization: a process that can be understood as health equity mainstreaming. Developing a research culture that delivers health equity-responsive research is seen as crucial to produce new knowledge that identifies the role that wider social determinants of health play in (re)producing inequalities. This knowledge then can be used to inform and innovate policy and practice to reduce these inequalities.

In this paper we describe CLAHRC NWC’s initiative that aimed to mainstream a focus on health equity and to assess the impact on organizational culture, research processes and individual research practice. The paper proceeds as follows. We begin by describing the function and structure of CLAHRC NWC. Then we introduce the analytical approach we adopt. This will be a combination of two bodies of literature: gender mainstreaming, which provides a framework to explicate “what is to be done” to begin a process to institutionalize mainstreaming in a research organization, and Extended Normalization Process Theory (ENPT), which allows us to examine “how things have to be done” while taking into account the specific contextual factors which promoted or frustrated these efforts. These frameworks will influence our definition of health equity mainstreaming. We conclude by emphasizing the need for a robust strategy for mainstreaming a focus on health equity as an important building block for creating transformative change in applied health research, in policy and practice, and amongst research funders. The inequalities exposed by COVID-19 are a timely reminder of the need to integrate a routine health equity focus in research that could unveil “context-specific factors related to real world health program, policy and system decision” as well as “the negative impact of implementing new interventions or technologies on health inequalities” [12]). Though findings are focused in the United Kingdom, there are implications for anyone concerned with putting health inequalities centre-stage in the research agenda.

A note on language. In this article we choose to use the concept of health inequalities following the usual practice in the United Kingdom. We understand health inequalities as the avoidable, unfair and systematic differences in health status, quality of care and access to opportunities between different groups of people [49]. Health inequalities arise from a complex and unequal interaction of many socioeconomic factors including, housing, income, education, social isolation, disability—all of which are strongly affected by one's economic and social status [23]. We refer to these factors as the upstream social determinants of health [18].

### The organizational context

CLAHRC NWC is one of 13 CLAHRCs funded by the NIHR from 2014 to 2019. It is organizationally diverse, including three universities, the Innovation Agency NWC, five National Health Service (NHS) organizations, nine Local Authorities (LA) and 17 NHS acute, mental health and community trusts. In addition, 170 members of the public were registered as Public Advisers (PA) and involved in all aspects of the programme. CLAHRCs aimed to support the translation of research findings into practice to improve population health. In common with other CLAHRCs, CLAHRC NWC shared the commitment to coproduction, public involvement and capacity development. However, its distinctive aim was to ensure that everything it did had clear relevance and utility for action to tackle the root causes of health inequalities (NIHR CLAHRC NWC 2013). The scale of health inequalities in the North West of England was a major factor influencing this emphasis [50, 51].

**Fig. 1:**
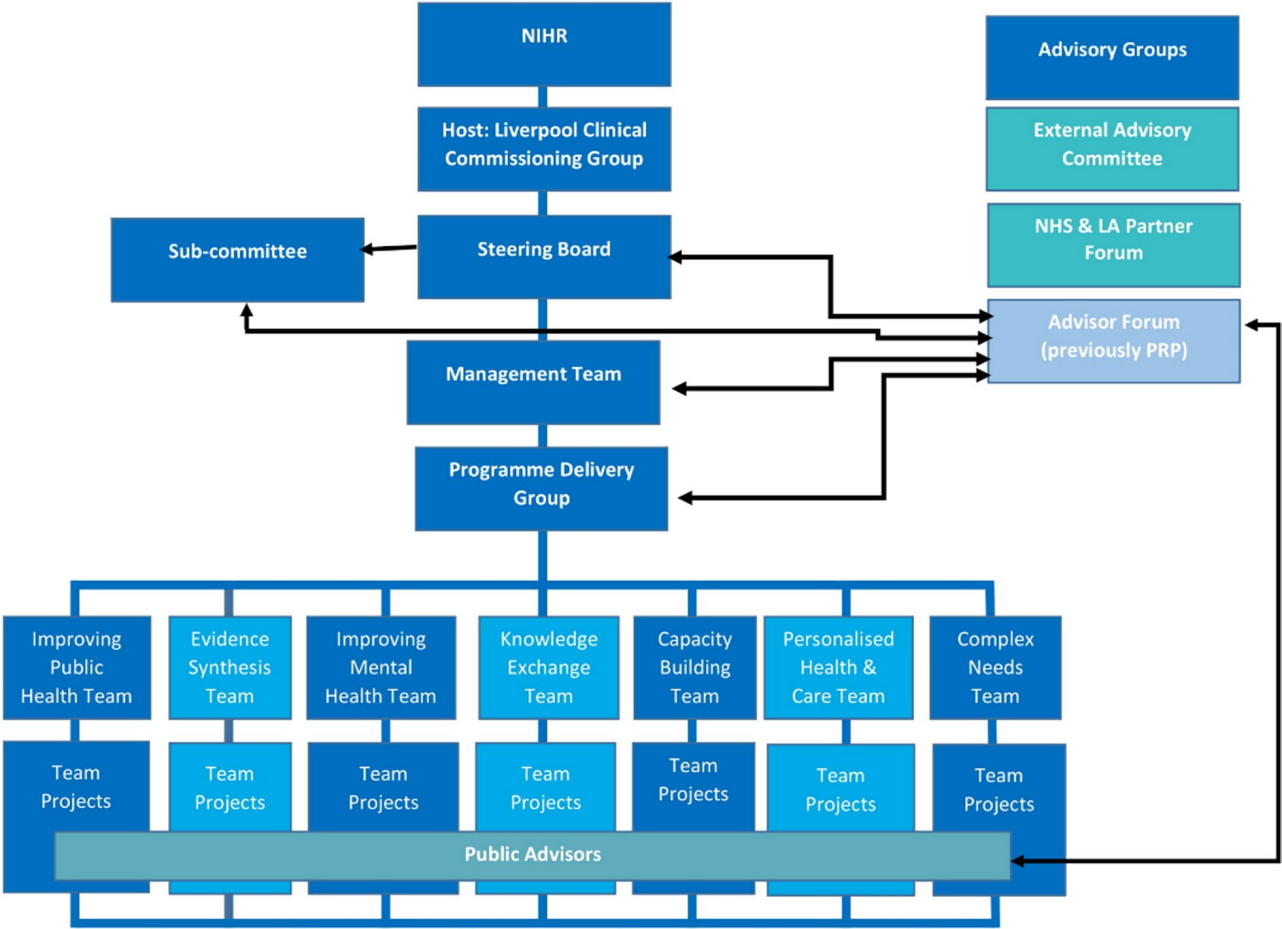
organizational structure of NIHR CLAHRC NWC

The organizational architecture of CLAHRC NWC is shown in Fig. 1. The Steering Board (SB) included representatives from the NHS, LAs, university partners and PAs, with an independent chair. A subcommittee of the SB reviewed project proposals and made recommendations on funding. The management team comprised the Director, Programme Manager, Operations Manager, Director of Engagement, Director of Capacity Development and Theme Leads. There were four thematic programme and three crosscutting Themes. In addition, an Advisers Forum, open to all members of the public registered as PAs, oversaw the public involvement policy and sent representatives to the SB and the CLAHRC management group.

### Conceptualizing mainstreaming

The Cambridge Dictionary [5] defines mainstreaming as a “process” whereby something becomes “accepted as normal by most people”. In this paper we combine ENPT and gender mainstreaming literature, as we believe this may give a more nuanced analysis of the processes, relationships and factors through which health equity mainstreaming is implemented and contested in large research organizations.

 ENPT [24] enables one to explain how new ways of thinking become routinely embedded in design, evaluation and implementation processes and in organizational practices. The emphasis is on the interplay between context and emerging expressions of agency. An enabling context is theorized to have two elements: capacity and potential. *Capacity* refers to the social, structural, material and cognitive resources available and includes making explicit and systematic the values of the organization, rules, roles of people involved, practices and perspectives. *Potential* refers to people’s readiness and commitment to act. In addition to an enabling context, a successful Normalization process requires collective and individual agency to be activated in the form of *capability* and *contribution*. Enhanced *capability* requires resources (including conceptual frameworks) to be “workable” i.e. easily integrated into existing routines and structures. *Contribution* depends on individuals’ commitment. For individuals to mobilize resources to make things happen, they need to make sense of new knowledge and skills, and recognize their legitimacy and benefits. Factors such as number and size of organizations also play an important role in shaping the success of Normalization processes [27].

It is worth noting that May’s examples [24] and the bulk of other uses of the ENPT are located within health-care organizations and systems. In contrast, we have applied the theory and concepts within research organizations and systems and in particular within a complex partnership-based organization to understand the factors shaping the implementation and embedding of new ways of thinking, enacting and organizing practice inherent in the equity mainstreaming process.

*Gender mainstreaming* gained worldwide visibility after the Fourth World Conference on Women in 1995. It has emerged as “a strategy for combating gender inequality in the long term” [4, 40] endorsed by international agencies such as the United Nations Development Programme (UNDP), the European Commission, the Commonwealth Secretariat, the World Bank and WHO. Like other concepts widely used in policy (e.g. empowerment) [6], the mutability of the term *mainstreaming* has allowed it to be translated into diverse political contexts and take on a range of divergent meanings [38], but according to the original conceptualization it is:

1. A strategy for action to achieve equity by removing biases and injustices (Woodford-Berger [54]).
2. A process that aims to transform ways of thinking and acting as well as organizational structures that are equity-blind or sustain existing inequalities.
3. A capacity-building and assessment approach to integrate equity issues into all the activities funded and/or executed by an organization [40].
4. An approach that seeks to diffuse responsibility for integrating an equity perspective beyond specialized units/teams through training, guidelines, checklists—“making it a routine concern of every bureaucratic unit” [26]: (ii) and everybody’s business.

There have been a number of attempts to incorporate action to address health inequalities across organizational policies and practices [28–30, 47, 52]. However, our rapid review combining searches from Google Scholar, Google (to identify grey literature) and the databases of MEDLINE and PubMed did not find an explicit definition of health equity mainstreaming, nor did we identify any initiatives that sought to embed a health equity focus across research organizations. These findings are confirmed by a forthcoming review of English-language papers/resources aiming to strengthen the equity focus in health research, which has found that, with notable exceptions [12, 35], published evidence on the processes and effectiveness of attempts to integrate a health equity focus across research organizations is lacking (Halliday et al. personal communication). Finally, our definition of health equity mainstreaming draws heavily on ENPT and gender mainstreaming literature and understands mainstreaming as a strategy to influence the implementation, integration and institutionalization of ways of thinking and acting to tackle the root causes of health inequalities [13, 42–45].

## Methods

### Context

An internal evaluation of CLAHRC NWC was conducted in 2017/2018 to assess the extent to which three strategic objectives on (i) public and stakeholder involvement, (ii) embedding a health equity focus and (iii) research capacity building were achieved. The study was conducted by teams of academics and PAs. The evaluation addressed the overall performance of four linked programmes: the Public Health research (PH) programme involving participatory research in 10 neighbourhoods; the Partners’ Priority Programme (PPP) involving evaluative research on new models of care; the Intern programme (IP) providing research training for NHS and local government staff, and the extent to which strategic objectives for public and stakeholder involvement, health equity and research capacity building were achieved across CLAHRC NWC. The findings presented in this paper relate to the achievement of the strategic objective of embedding a health equity and are based on data from across the four work programmes.

### Data collection and analysis

In addition to data from interviews and focus groups, the evaluation collected data from internal documents (e.g. policies, strategies and minutes of management and SB meetings), monitoring data and data from feedback forms completed by people using the Health Inequalities Assessment Toolkit (HIAT). Data were collected from 131 individuals via face-to-face interviews (*n* = 58) and focus group /workshops (*n* = 73). These included staff from CLAHRC NWC’s NHS, local government (LA), university and third-sector partners; PAs; and professional interns supported by CLAHRC NWC. Information sheets and consent forms emphasized that participation was voluntary.

As each component of the evaluation had its own objectives, the interview and focus group topic guides varied in the extent to which they prompted research participants about the strategic objectives, but all collected relevant qualitative data. We use ENPT and Moser and Moser’s work [28] as analytical frameworks. ENPT provides the tools to explain the “social processes” [25]) that promoted or frustrated health equity mainstreaming efforts. Moser and Moser’s work [28] provides the stages to map progress towards health equity mainstreaming and the factors that promoted or frustrated these efforts. We were aware that UNDP [45] and the United Nations Evaluation Group (UNEG) [46], amongst others, make an explicit distinction between institutional and programmatic mainstreaming and provide a list of indicators. However, we consciously chose Moser and Moser’s work as the main analytical framework because their stages to measure progress are an amalgamation of institutional and programmatic strategies. This made the evaluation process more manageable. As we mentioned earlier, the goal of the evaluation was to assess progress in relation to three strategic objectives, being health equity one of them. It would have been impractical to employ all the categories and indicators stipulated by UNEG [46] and UNDP [45] to collect and analyse the data.

As the analysis evolved, other themes and codes were added [15]. Researchers first familiarized themselves with the data by reading the transcripts, noting new themes. The final coding frame was then systematically applied to all transcripts. The coding frame was uploaded to Excel and data were coded into a set of analytical charts. These charts were studied to identify common or divergent perspectives and the main authors discussed potential explanations and interpretations. A PA panel took part in two workshops to discuss data interpretation and preliminary findings. Content analysis of CLAHRC NWC policies, strategies, reports, minutes of the SB, and feedback forms HIAT assessments were also conducted to identify references to health inequalities.

Where quotations are used to illustrate findings, the reference includes (i) the data collection method with a unique number (int14 = interview n.14; grp = focus group; HIAT feedback form), (ii) respondent’s organization (Local Authority = LA; NHS; Public Adviser; Academic; Intern); and (iii) the evaluation component (PH = Public Health programme; PPP =  Partners’ Priority Programme; Intern programme = IP; CC = CLAHRC strategic objectives). On occasion we have used research participants’ direct short verbatim words or expressions in the text to convey meaning about feelings or situations. These words are not fully referenced, to make reading more agile, but are italicized to be easily differentiated from the authors’ interpretation. This style follows common practice in the field of anthropology and ethnographic writing.

### Ethics

Ethical approval for primary data collection was obtained from the university where the lead researchers were based: Lancaster University for research on the Public Health (PH) programme and CLAHRC NWC strategic objectives (CC); Liverpool University for research on the Partners’ Priority Programme (PPP); and University of Central Lancashire for research on the Intern programme.

### Results

The aim of this section is to discuss the three stages used to measure progress towards equity mainstreaming. For ease of analysis we present these stages in a linear fashion although they were iterative.

For any attempt to mainstream health equity to have far-reaching and lasting consequences on research practice, it must first create institutional-level changes. Yet, institutional-level change is stubbornly difficult and can take years [20]. Additionally, as CLAHRC NWC was a multi-agency collaboration, these changes had to impact on multiple diverse organizations. Nevertheless, the examples of change across institutional systems and processes we identify in the next two subsections show overall progress.

### Adopting a conceptual framework that foregrounds health equity

A major requirement for progress in mainstreaming is the development and adoption of a conceptual framework that foregrounds the issue being addressed, in this case, health equity [38], [46]). An analysis of CLAHRC NWC formal documents showed that attempts were made at an early stage to define the concept of health inequalities to be adopted in the organization. Although the word “mainstreaming” was not explicitly used, there were clear statements about the importance of, and commitment to, embedding a focus on action to reduce health inequalities in the organizational architecture of the CLAHRC NWC including structures, processes and projects.

Key examples of these statements are found in the original funding proposal submitted in 2013, the website and promotional materials. These emphasized the collaboration’s commitment to “*produce applied health research that contributes to tackling health inequalities through improvements in public health and chronic disease*” [32]). The concern with health inequalities came into focus with the funding proposal’s acknowledgement that the NWC has one “*of the most striking variations in health and wellbeing in England*…” [32]). The proposal went on to argue that health equity would be a crosscutting issue and a CLAHRC-wide responsibility. It identified theme management as the primary site for monitoring and assessing the impact of activities on inequalities:

*Each Theme will have a Theme strategy committee (TSC) chaired by the Theme leader and comprising Theme managers (...) The TSC will be responsible for (…) assessing the impact of the projects on health inequalities and patients outcomes*. (CLAHRC NWC Full application to NIHR, p.20)

Despite the prominence of these statements, two interlinked factors potentially diluted the message that addressing the upstream social determinants of health inequalities was a CLAHRC-wide responsibility. First, the location of the message in the funding proposal may have been problematic. Work on health inequalities was described within the Public Health Theme (Fig. 1), potentially suggesting that it was the primary responsibility of this theme. Whilst this positioning was argued to be a response to the emphasis the funder placed on a thematic structure for the programme, it would have been possible to locate health inequalities as a crosscutting theme and in doing so it would have helped to build a shared sense of accountability across CLAHRC NWC. Additionally, whilst health inequalities were mentioned at several other points in the funding proposal, the prominence varied significantly across the descriptions of specific themes. Second, whilst a policy on public involvement and a strategy for capacity building were produced, there was no explicit strategy or policy on how the focus on reducing health inequalities would be mainstreamed across the CLAHRC NWC. We will discuss the implications of these factors in the next section.

Initially, CLAHRC NWC took three important practical steps in pursuit of the health equity objective. First, it appointed senior staff with an international track record of work on health inequalities, to take responsibility for the mainstreaming agenda from the point the original funding bid was developed. Second, it articulated an explicit definition that recognized that inequalities in health cannot be tackled without fully understanding and addressing their upstream social determinants. This marks a shift from the dominant framings of health inequalities in the health sector as individualized “lifestyle-centric” to recognize how “*organization and structural factors are the cause of social inequalities that affect health outcomes*” [32]. Third, in 2015, CLAHRC NWC co-produced the HIAT to support researchers and others to assess the extent to which planned activities were sensitive to health inequalities [37].

The HIAT further highlighted CLAHRC NWC’s emphasis on the upstream social determinants of health inequalities. However, seeking to embed a conceptual framing of health inequalities that was sensitive to social inequalities, public involvement and coproduction across all CLAHRC NWC activities and within partner organizations raised several challenges discussed in the next section.

### Developing structures for embedding health inequalities awareness

The literature on gender mainstreaming highlights institutional commitment to develop relevant “capacity” as another indicator of progress [33]. Analysis of documents and discussions with research participants revealed that CLAHRC NWC invested considerable resources in strengthening its infrastructure to support capacity in relation to the mainstreaming of a health equity focus in all its work. For example it:

- Invested in specialized staffing to support partners staff to embed a health equity perspective across all levels of the organization and its portfolio of research and related activities.
- Provided routine training and individual bespoke advice to all staff, PhD students and PAs.
- Partnered with professionals from other regional and national agencies to advance the goal of mainstreaming health equity beyond CLAHRC NWC.
- Allocated a dedicated budget for training, dissemination activities and the development of resources such as a website, training materials and accessible HIAT leaflets.

CLAHRC NWC also sought to strengthen the degree of transparency and accountability through reporting and monitoring processes. For instance, the SB endorsed mandatory HIAT assessments for all activities seeking funding support from CLAHRC, including interns and PhD students. In addition, the SB requested that quarterly progress report templates be modified to include a section for reporting on the extent to which a focus on health inequalities had been integrated into activities.

### Implementing health inequalities mainstreaming across CLAHRC NWC activities

In what follows we describe the results of the practical application of health equity into all activities and all stages of the programme: health inequalities sensitization; mainstreaming through toolkits and reporting; achieving better health equity-sensitive evaluations; fostering local collaborations that include practitioners and community members to address social inequalities in health; valuing public involvement; and achieving reductions in health inequalities. It is worth noting that given the short time frame of CLAHRC NWC and the complexities involved in tackling health inequalities, we did not expect to find examples of an impact of mainstreaming on reducing health inequalities. Nevertheless, the examples provided, described by a university staff member “*as oases in a desert*”, provide a ray of hope.

(i) Health inequalities sensitisation
  Compulsory HIAT assessments, one-to-one support, specialized training sessions, informal learning opportunities, dissemination events, public engagement activities, resources (such as quizzes and games) and participation in research projects all contributed to increased awareness amongst partners, PAs and the wider public about the social and economic causes of health inequalities. Research participants described HIAT assessments as “*triggering a light bulb moment*” and “*broadening horizons*” by revealing how ill health is linked to socioeconomic factors. Others mentioned that training was “*transformational*” because it challenged the notion that health inequalities are beyond professionals’ remit and helped them to recognize “*that health inequalities is not the responsibility of professionals specialized on health inequalities; it is everybody’s business*”. PAs also stated that CLAHRC NWC helped to create an environment that normalized discussions around health inequalities.
(ii) Mainstreaming through toolkits and reporting
  As noted earlier, HIAT assessments of all activities and regular reporting on health inequalities were mandatory. A subcommittee of the SB reviewed all HIAT assessment reports and gave feedback on how to improve the health equity focus of proposed work. Proposals that failed to complete the HIAT were rejected. Some respondents described the emphasis on assessments and reporting as a way to remind people that health inequalities are “*everyone’s responsibility*”. Others described this obligation as a “*carrot-and-stick*” approach that was important because “*academics wouldn’t have used it otherwise*”. There were multiple comments about the benefits of the HIAT assessment process. As one intern noted:
  *The health inequalities assessment toolkit was great. That was all new to me and very useful, and that thinking upstream stuff, it was a lot of food for thought. I felt like my brain was running out of my ears, to be honest, but it was really good*. (CB.int.008, Intern)
  Similarly, an NHS partner involved in evaluating a new model of care commented that:
  *Rather than simply thinking about outcomes, the HIAT tool allowed us to think more effectively around the data we were collecting and how we were collecting it, as well as how we can interrogate the data to gain further insight around socioeconomic and demographic factors*. (HIAT feedback form, NHS partner)
(iii) Achieving better health equity-sensitive evaluations.
  As Sen et al. [38] argue, getting the right data and indicators is a prerequisite for more effective actions on health because: “*what gets measured is what gets done*”. Partners and PAs reported that HIAT training and assessments helped them to develop evaluations that were sensitive to health equity and enhanced their practice. For instance, several respondents agreed that in the NHS and LA, “*things get implemented, but nobody measures the impact of implementing something*”. And as this partner commented, evaluations sensitive to health equity brought to the fore issues of accountability and “*wise*” expenditure of public money:
  *I think probably we’ve conducted more robust evaluation than we would have done if we hadn’t been involved with CLAHRC (...) I think that’s helpful because it makes us consider whether what we’re doing is effective and how it can be changed rather than just keeping plodding on doing what we’re doing because we think it’s the right thing to do*. (ESK.int.190118, Academic partner)
  Most respondents stated that they had learnt about the importance of collecting disaggregated data by socioeconomic status and other relevant determinants of inequalities to measure any differential impacts of interventions. This senior CLAHRC NWC staff member emphasized this learning:
  *[Partners staff] the identification of the health inequalities and measurement has been real learning and real change, particularly around using disaggregated data (...) in undertaking their evaluations (...) They’ve had to look at how do we collect the data in that way, and that’s been real, real learning for them*. (PPP.fg.02, Academic partner)
  For one NHS partner, the realization that there were “*limitations of data coming*” from their organization was “*disappointing*”. Others conducting reviews found the data limitations of primary research “*frustrating*” and decided to report these gaps in their outputs. Through reporting, they hoped to make visible the need for disaggregated data in all research projects. Another team of NHS partners changed their organizational structures to get more health equity-sensitive information going:
  *quite a long way to adapt their current practices to design their data capture questionnaires that go right across their organization, not just for the evaluation but for the way that they record and track their service users, the disaggregated data, because they weren’t collecting it in that way before. So that’s quite a big service change for them to make, so they’ve been willing to take on board some of the ideas and suggestions and put them into practice*. (PPP.fg.02, NHS partner)
(iv) Fostering local collaborations that include practitioners and members of the public to address the social inequalities in health
  Developing collaborations between different agencies and with members of the public has been argued to be an effective way to address social inequalities in health [8]. Certainly many CLAHRC NWC staff and partners appreciated that “*joint work between universities and the service side*” opened opportunities to access resources like databases, tools and ways of presenting information, deepened their understanding of health inequalities and encouraged them to use the collaboration to rethink how they address health inequalities. Several academics particularly valued the opportunity to work with local government and organizations outside the traditional remit of public health, such as those in the fields of housing, environment and transport, as well as with third-sector organizations, community groups, residents, local businesses and local employers to address local social determinants of health. This university partner reflected on the impact of CLAHRC NWC on fostering a research culture of coproduction through collaboration with LAs:
  *For example, places like (LA name), they are trying to address debt, trying to bring in stuff like financial education type support. I think it has changed the dialogue and all the partners who have been involved, I do detect that*. (EKM.int.190118, Academic partner)
(v) Valuing public involvement
  In the field of gender mainstreaming, another indicator of success is the involvement of women or women’s rights organizations in the planning or formulation of programmes and the valuing of their knowledge and contributions [41]. By the same token, addressing health inequalities requires public involvement as an entry point to understand the perspective of those experiencing social and health inequalities. A number of interviewees and comments on feedback forms revealed that the HIAT assessment process helped some people to comprehend the importance of involving members of the public to design health equity-responsive research that will lead to a greater volume of evidence with the potential to inform effective interventions, as this academic highlights:
  *Well, I think that’s where public engagement and the HIAT actually mesh together, in that you can’t really do a HIAT without engaging with people, members of the public or patients or carers, because you’re turning the research topic round to what do they think would be helpful to them*. (EKM.int.240118, Academic partner)
  LA respondents made similar points, when asked whether they had benefited from being involved in the CLAHRC:
  *Yeah, I do very much so (...) I suppose some things I’ve learnt have been around, you know, when you’re working with communities actually trying to do something*. (PH.int.9, LA partner)

## Discussion: Understanding factors that enabled or prevented mainstreaming

We utilized May et al.’s ENPT to identify and explore factors associated with context and agency that enabled or impeded mainstreaming progress [24].

### Contextual factors that influenced mainstreaming

According to the theory, as explained earlier, contextual factors influencing mainstreaming processes include *capacity* and *potential.*

(i) Capacity
  The previous section demonstrated how CLAHRC NWC invested in developing structural and cognitive resources to facilitate the process of mainstreaming health equity. But despite these efforts, there were problems. Perhaps the most important problems emerged because of the lack of an explicit mainstreaming strategy, which resulted in a lack of rigorous systems for ensuring accountability and transparency. Given the lack of readily accessible literature, guidelines or examples on how to embed a focus on health equity at an institutional level or in research processes, and the short time available to produce the original funding bid, it is understandable that a strategy was not put in place initially. However, it is likely that this led to a lack of clarity about whose responsibility it was* “to integrate and coordinate work (…) to reduce health inequalities*” as set out in the original proposal. The importance of having a strategy that spells out “norms” or “rules” to give structure to meanings and define behaviours within organizations has been highlighted [25, 27]. Indeed, gender experts have noted that if mainstreaming is to be successful, organizations must make explicit its importance and deal with issues of accountability and roles: “[if gender] is not integrated from the outset of the process, it will structurally determine that…[it] does not receive necessary attention and priority throughout the remainder of the process” [1], see also [46].
  Insights from the literature suggest that if CLAHRC NWC had had an explicit strategy on health equity mainstreaming from the onset, it is more likely that the collaboration would have established a central “team” with a remit to foster accountability and transparency for health equity mainstreaming across the complex CLAHRC architecture, rather than locating this responsibility in the Public Health Theme. However, a senior university partner involved in writing the original bid argued that *“responsibility was contained within a theme to ensure it could be delivered in a focused rather than diffuse way with the most senior experts in control of the process who also had responsibility for engagement*”. From this perspective, problems arose not because of where the health equity team were located in the organization, but because, in practice, staff deflected responsibility. Particularly in the early years, routine data suggest that the dedicated staff member working part time on the health equity mainstreaming agenda was perceived as having primary responsibility for training, promoting and monitoring the implementation of a health inequity focus. It is likely that this process would have been compounded by the lack of an explicit strategy, which allowed the message that health equity mainstreaming was a CLAHRC NWC-wide responsibility to be diluted.
  Problems also arose because many of the first-phase project proposals began before the HIAT was in place, so a focus on health equity had to be “retrofitted”: there was perhaps an understandable reluctance amongst some research staff to engage in this process with enthusiasm. Third, the HIAT team was on a steep learning curve in terms of how the health equity mainstreaming objectives could be operationalized. This led to delays in the development and provision of cognitive resources (i.e. such as training materials, guidelines, checklists or case studies). In addition, it took nearly a year to appoint a senior researcher to lead on co-developing the HIAT and a further seven months to launch the first version of the tool in March 2015. However, this process itself was participative and involved a significant number of people (PA, partner professionals, academics, etc.) in a series of iterative co-development and review meetings focused on the tool itself and related web resource. These meetings thrashed out many disagreements and concerns about definitions, emphasis on social determinants, and expectations enabling the developing of a more accessible and appropriate tool.
(ii) Potential
  Potential is the “readiness” to act, embrace new knowledge or adopt a new practice which is, in turn, highly dependent on what people already know (cognition). Pedraza-Fariña’s study [34] on social innovation within collaborations emphasized the impact of cognitive distance between people—i.e. the gulf between different ways of acquiring knowledge and understanding information—which “*can prevent fruitful idea recombination*” and collaboration. In essence, cognitive distance hampers people’s potential to engage with other ways of knowing, creating conflicting perceptions of what counts as evidence and what problems and approaches are worthy, rigorous and feasible [19, 34].
  Though not explicitly referenced, forms of cognitive distance were one of the most frequently mentioned barriers to partners’ staff engagement with CLAHRC NWC’s approach to health equity during the first 18 months. For instance, while there was widespread agreement that health inequalities were important, there were disagreements over the centrality of the upstream social determinants in CLAHRC NWC’s approach to health equity, and even, as this partner noted, disagreement about how prominent the health inequalities focus should be:
  *I don’t know, but some very senior people have said “we would like you to tone it down next time because other people are complaining, saying, ‘bloody Health Inequalities it’s figuring all the time; I just want to answer a research problem; why have we got to worry about that?’”* (APE.int.190118, Academic partner)
  There were also different understandings of the concept of health inequalities. This participant explained how professionals struggled to integrate CLAHRC NWC’s focus on the upstream social determinants of health inequalities as opposed to a disease focus into their pre-existing projects and activities:
  *Part of the reason why I have struggled a bit trying to explain to them because people tend to think about like health, “well I’ve got cancer” or “my friend or my family’s got cancer”, so it's a real physical or health problem or somebody’s got dementia or severe depression or whatever, but it’s all these sort of like precursor still a lot of these things I see*. (EKM.int.190118, Academic partner)
  Data from the interviews suggest that this resistence could also be driven by ideas about the limited benefit professionals would obtain from engaging with health inequalities, echoing research that suggests that cognitive distance is also shaped by professional self-interest [38]. For instance, those reluctant to invest time to retrofit health inequalities in their existing projects claimed that HIAT assessments and progress reports were too “*restrictive*”, “*bureaucratic*” or “*unnecessary*”.
  Finally, people’s potential to act is dependent upon pre-existing relationships [21, 34]. CLAHRC NWC brought together organizations and individuals from very diverse disciplines and backgrounds, most of whom had no previous connecting ties. This can have serious implications for the levels and extent of trust, which is an important requirement for cooperation [10].
  *We bring a group of people together that have not worked together before, and that was a major challenge. So you’ve got a lot of money to deliver something really quite big among a group of people that have no track record of working together before, and that was a real stress (...) it really impacted on efficiency and the ability to deliver something for quite a long time*. (FW.int.090218, Academic partner)
  The success of one stream of work, the PPP, illustrates the importance of cognition and pre-existing relationships to activating people’s predisposition to engage with health equity mainstreaming. This programme was established in 2015, within the Knowledge Exchange Theme, to evaluate new models of care. Widely perceived to be very valuable, respondents' comments suggest that a key ingredient in this was the programme lead’s commitment that enthused members of her team. With a background in public health, she advocated for action on the upstream social determinants of health inequalities and ensured that health equity mainstreaming was a crosscutting goal in the programme. She was also an “in-betweener”. As an academic and local government practitioner, she spoke two “languages”, so she understood and helped to bridge different epistemic worldviews. There were of course other dynamics at play, notably the fact that this programme was established at the request of NHS and local government partners and had the SB’s approval. This was instrumental in legitimating and facilitating the lead’s attempts to make health equity a priority.

### Emerging expressions of agency that influenced mainstreaming

Enabling what May et al. [25] term “emerging expressions of agency” is essential to normalizing new ways of thinking and acting. These emerging expressions of agency involve *capability*, which requires that resources are “workable” so that they can be easily integrated into existing routines and structures, and *contribution*, which happens when individuals become active participants in mobilizing resources to normalize practices.

(i) Capability
  As argued earlier, different understandings of health inequalities played a role in determining whether people resisted or engaged with health equity mainstreaming. But sometimes it was a lack of confidence, and not a lack of desire and knowledge, that prevented people from designing health equity-sensitive activities and/or supporting others to do this. As CLAHRC NWC developed more training and developmental sessions on health inequalities, the general perception was that knowledge of, and confidence in, using the HIAT tool—making it more “workable”—grew over time. As one respondent explained:
  *I think, when I was looking at it just as a tool without a project to apply it to (...) I mean I could understand the words that I was seeing on the page but I couldn’t imagine how it would be applied in actuality (...) so that whole process of, look at the HIAT tool, apply it to a project, help them with the project and then get some feedback from (facilitator name) and then go around that again, that iterative process with (facilitator name’s) feedback I think has been a really important learning opportunity*. (PPP.fg.02, NHS partner)
  Once the HIAT tool was perceived to be workable, it was easier for people to see how it could be integrated into everyday practice. One postgraduate student, for example, highlighted how, after receiving training, they planned to use the HIAT in the future. Another student noted that applying the HIAT helped them to recognize the responsibility of all researchers in applying a health equity lens to health service research.
(ii) Contribution
  Positive contribution leading to integration of new practices is not necessarily the sum of potential, capacity and capability. As already noted, for some people, capacity-building activities and the requirement for HIAT assessments and reports were not enough to bring about shifts in thinking, which in turn impacted on people’s readiness to engage with health equity and the HIAT. Knowledge, perceptions about professional gain, lack of time to realign projects, lack of support within organizations, pressure to get on with the research and publish, and the weight of the mind-set that “this is the way we do things” were all factors that thwarted *contribution*: the mobilization of resources to normalize practices.
  On the other hand, there were many involved in CLAHRC NWC who accepted that health equity mainstreaming was a CLAHRC-wide responsibility. Their attitudes, combined with access to training and resources (structural and cognitive), helped them to become active supporters of practices that normalized a health equity focus into their own and their teams’ work. They showed a great attachment to CLAHRC NWC’s approach and became HIAT champions, playing a fundamental role in creating an environment to motivate others to engage with issues of health inequalities. As one core CLAHRC NWC staff commented, learning to implement the HIAT had been “*fantastic*” not only because it enhanced her own knowledge and skills but because it could enthuse and support others to use the tool. Similarly, an LA partner commented on how their expanding understanding of health inequalities affected their approach to data analysis and collaborations:
  *A lot of the broader health and equality stuff has probably affected how I look at data in other parts of the county. For example, I do quite a lot of work in (place name) working with one of the local GPs and a team of partners and community members looking at how we tackle some of the entrenched issues there. So that side of it probably has stepped back in, yes*. (PH.int. 01, LA partner)

### Strengths and limitations of the CLAHRC evaluation

CLAHRC NWC invested resources in cash and kind in conducting an internal evaluation, and a wide range of stakeholders—professionals and public—contributed a valuable diversity of perspectives to the interpretative process. The fact that the evaluation was conducted by an internal team enabled its members to navigate the intricacies of this complex collaborative organization and to draw upon embodied and tacit knowledge of the context in which CLAHRC NWC operated. This helped to fill gaps in the data and enabled access to a range of secondary data.

At the same time, however, this “insider position” can be viewed as a limitation. Several steps were taken to reduce “direct bias” [16] from CLAHRC NWC staff conducting the evaluation. These included: avoiding allocating interviews to members of the evaluation team with personal contact with the interviewee; initial transcripts coded by two of the evaluation team researchers and results compared; and a collective, iterative process of reflecting on data analysis and interpretation. PAs also reviewed a sample of transcript extracts to ensure that a public perspective of key themes informed the findings.

Finally, there were two limitations in the data we collected. First, there were some differences in the data collected from the different programmes of the evaluation. As each programme had its own objectives, the interview and focus group topic guides varied in the detail to which they prompted research participants about health inequalities. However, together they provided a rich picture across the collaboration’s work. Second, as that this was a qualitative study, we do not feel that our data allows for a robust detailed analysis of the differential impact of the mainstreaming activities across groups and work strands within the organization. The only area where we felt able to make “claims” relating to the scale of impact was in relation to the Partners’ Priority Programme. However, this should not be interpreted as meaning equity was more strongly mainstreamed in this programme compared to the other thematic areas of work.

## Conclusion

The findings reported here contribute to the literature on health equity in a number of ways. They provide insights into CLAHRC NWC’s attempt to bring a focus on health inequalities centre-stage by embedding it in its organizational culture, at all levels and in all processes and activities within this large and complex collaboration, a process that we define as health equity mainstreaming. The rationale of equity mainstreaming was not to have a direct impact on improving population health and health inequalities, but rather it was to develop a research culture and research practices that had health equity at its heart, maximizing the potential for the evidence produced to inform and innovate policy and practice to tackle these inequalities.

The analytical purchase provided by the use of these two frameworks in combination has illuminated important progress made in this endeavour, and the majority of respondents perceived that the focus on health equity has added value to their work and that of the collaboration. However, the attempt to mainstream a health equity focus has also been contested and has involved a steep learning curve for all involved.

Insights from the gender mainstreaming literature have provided a novel perspective on “what is to be done” to mainstream an equity focus across a research organization to support the design and implementation of research with enhanced potential to reduce health inequalities. This literature provided a framework through which to examine the nature and impact of structures, processes and activities put in place by CLAHRC NWC. However, recent scholarship on gender mainstreaming has shown that assessing progress calls for a rigorous understanding of “how things have to be done”, rather a than a single focus on whether a predictable set of stages have been meet. Here the application of Extended Network Theory was helpful in illuminating how specific contextual factors and dynamics can enable or hinder attempts to normalize a health equity perspective.

In particular, successful mainstreaming requires clarity and transparency about roles, responsibilities and accountability mechanisms for integrating and monitoring this focus. It will also require participation so that these responsibilities are widely distributed across an organization, marking, as a recent article on gender equity in science argued, an important shift from the measurement and sensitisation revolution to “the accountability revolution” [17] whereby equity becomes everybody’s responsibility.

## Data Availability

For confidentiality reasons, and due to the nature of the consent obtained, the qualitative interview transcripts cannot be shared. For further information related to this data set, please contact the corresponding author.

## Abbreviations

CC: CLAHRC strategic objectives
CCG: Clinical Commissioning Groups
CLAHRC NWC: Collaboration for Leadership in Applied Health Research and Care North West Coast
Fg: Focus group
HIAT: Health Inequalities Assessment Toolkit
Int: Interview
IP: Intern programme
LA: Local Authorities
ENPT: Extended Normalization Process Theory
PPP: Partners’ Priority Programme
PA: Public Advisers
PH: Public Health research programme
SB: Steering Board
NIHR: National Institute for Health Research
UNDP: United Nations Development Programme
UNEG: United Nations Evaluation Group
WHO: World Health Organization
